# Concurrent Phosphorus Recovery and Energy Generation in Mediator-Less Dual Chamber Microbial Fuel Cells: Mechanisms and Influencing Factors

**DOI:** 10.3390/ijerph13040375

**Published:** 2016-03-28

**Authors:** Abdullah Almatouq, Akintunde O. Babatunde

**Affiliations:** 1 Hydro-Environment Research Centre, Energy and Environment Theme, Cardiff University School of Engineering, Queen’s Buildings, The Parade, Cardiff CF24 3AA, UK; BabatundeA@cardiff.ac.uk; 2 Kuwait Institute of Scientific Research, P.O. Box 24885, Safat 13109, Kuwait

**Keywords:** bio-electrochemical system, phosphorus, phosphorus recovery, microbial fuel cell, struvite

## Abstract

This study investigated the mechanism and key factors influencing concurrent phosphorus (P) recovery and energy generation in microbial fuel cells (MFC) during wastewater treatment. Using a mediator-less dual chamber microbial fuel cell operated for 120 days; P was shown to precipitate as struvite when ammonium and magnesium chloride solutions were added to the cathode chamber. Monitoring data for chemical oxygen demand (COD), pH, oxidation reduction potential (ORP) and aeration flow rate showed that a maximum 38% P recovery was achieved; and this corresponds to 1.5 g/L, pH > 8, −550 ± 10 mV and 50 mL/min respectively, for COD, pH_cathode_, ORP and cathode aeration flow rate. More importantly, COD and aeration flow rate were shown to be the key influencing factors for the P recovery and energy generation. Results further show that the maximum P recovery corresponds to 72 mW/m^2^ power density. However, the energy generated at maximum P recovery was not the optimum; this shows that whilst P recovery and energy generation can be concurrently achieved in a microbial fuel cell, neither can be at the optimal value.

## 1. Introduction

Microbial fuel cells (MFCs) are systems that convert chemical energy in an organic substrate in wastewater into electrical energy. Phosphorus (P) is one of the key pollutants that can be removed during wastewater treatment in MFC with the concurrent generation of energy. P is essential for crop growth, and approximately 60% of P used around the world comes from non-renewable sources [1]. Therefore, there is increasing interest in finding new sustainable sources of P. Wastewater is a rich source of nutrients that can be used as a sustainable source of P. Magnesium ammonium phosphate hexahydrate (MAP or struvite) is an efficient slow release fertilizer [2]. The mechanism of struvite precipitation is highly dependent on solution pH (pH > 8). The precipitation occurs in equimolecular concentration of magnesium (Mg), ammonium (NH_4_) and P; and these combine with water to form struvite [3]. P can be recovered as struvite from different waste streams including reject wastewater and digester effluent; and it can be achieved using several methods, including: chemical addition, carbon dioxide stripping or electrolysis [4,5,6]. However, the chemical addition is a costly process and the chemicals used to raise the pH can account for up to 97% of struvite cost [6,7].

Recent research findings on MFCs have demonstrated their potential to recover P as struvite [7,8,9]. P can be recovered exclusively through precipitation in MFC because P compounds are not involved in electron transfer via reduction—oxidation (REDOX) reactions [10]. P recovery as struvite using MFCs involves cathode reactions, whereby water is consumed and hydroxide is generated due to electrons transferred from the anode to the cathode (Equations (1) and (2)):

(1) $$\mathrm{Anode}\mathrm{chamber}:{\mathrm{CH}}_{3}{\mathrm{COO}}^{-}+4H_{2}O\to 2\mathrm{HC}O_{3}^{-}+9H^{+}+8e^{-}$$

(2) $$\mathrm{Cathode}\mathrm{chamber}:2H_{2}O+O_{2}+4e^{-}\to 4{\mathrm{OH}}^{-}$$

The generated hydroxide leads to increase in the pH around the cathode, and P starts to precipitate. P recovery from different wastewater sources has been widely studied using single chamber MFCs [5,8,9]. Ichihashi and Hirooka [9] reported 27% P recovery as struvite from swine wastewater. It was noted that precipitates formed around the cathode, which indicates that the pH around the cathode is higher than at any other place in the MFC. In addition, synthetic wastewater was used in a single chamber MFC to identify the effect of Mg and NH_4_ concentration on P recovery as struvite [8]. Dosing ammonium and magnesium for struvite precipitation is essential to form struvite; as the concentrations of NH_4_ and Mg increases, more P is precipitated. In another study, a 3-stage single chamber MFC was used to recover 78% P as struvite from human urine [4]. Using urine as a substrate increased the recovered P because urea hydrolysis increased the pH and created an alkaline environment which accelerated the precipitation. From these studies, it has been shown that MFC has the potential to recover P as struvite from different wastewater sources. However, most of these studies were conducted in single chamber MFCs, where pH buffering may occur due to the accumulation of protons and hydroxide ions in the same electrolyte; this could limit the P recovery. To overcome this limitation, dual chamber MFCs are increasingly being used. They have better separation between the anode and cathode chambers; this leads to pH splitting which creates an alkaline environment around the cathode to improve the P recovery process [10]. Such a 2-stage dual chamber MFC was operated in this study to recover P in the form of struvite. The specific objectives of the study were: (i) to understand the mechanisms of P recovery as struvite in dual-chamber MFC; and (ii) to identify the influencing factors for the P recovery.

## 2. Materials and Methods

### 2.1. MFC Configuration

**Figure 1:**
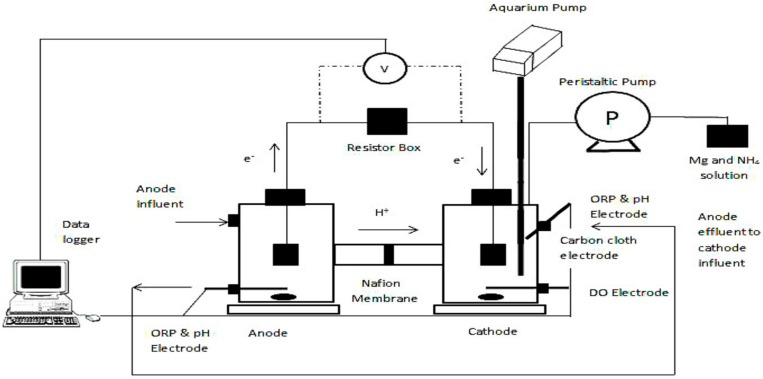
Experimental set-up of the dual-chamber MFC.

Two sets of dual-chamber H-type bottles (Adams & Chittenden Scientific Glass, Berkeley, CA, USA), were used to construct the MFCs (see Figure 1). 

Anode and cathode electrodes were made of carbon cloth 2.5 × 5 cm (projected area of 25 cm^2^) with a volume of 300 mL for each chamber. The cathode contained a Pt catalyst (0.5 mg/cm^2^ 10% Pt on Carbon Cloth Electrode) to improve cathode performance, whilst the anode is plain carbon cloth. Both electrodes were connected with a titanium wire (0.5 mm, purity > 99.98%, Alfa Aesar, Heysham, UK). A Nafion membrane (Nafion 117#, Sigma Aldrich, London, UK) was placed in the middle of the anode and the cathode. The membrane was pre-treated by boiling in H_2_O_2_ (30%) and deionized water, followed by 0.5 M H_2_SO_4_ and deionized water, each for 1 h, and thereafter it was stored in deionized water prior to being used. The MFCs were maintained at 20 °C in a temperature controlled room.

### 2.2. MFC Experimental Design and Start-Up

The anode chambers were inoculated with a 1:1 mixture of activated sludge obtained from a wastewater treatment plant in Cardiff and anolyte medium (mainly containing acetate). The external resistance was 1000 Ω at the initial stage of the operation, and it was reduced to 430 Ω where the system produced the maximum power in the polarization test. During the start-up-period, the cathode chamber was filled with 50 mM phosphorus buffer, and it was continuously aerated using an aquarium pump. Once the MFC achieved stable electricity production for at least three cycles, the phosphorus buffer was replaced with anolyte effluent from another batch.

The MFCs were operated in 2-stage feed-batch mode at room temperature. In the 1st stage, the synthetic wastewater was fed to the anode chamber for COD removal. At the end of each cycle, the effluent from the anode chamber was filtered and directed into the cathode chamber (2nd stage) for P recovery (see Figure 1). Anode influent pH was adjusted to pH = 7 and an aquarium pump was used to supply air continuously to the cathode chamber in order to provide oxygen as electron acceptor. Media replacement was carried out in a glove box to maintain the anaerobic environment for the anode biofilms. The synthetic wastewater contained 1 g/L sodium acetate; KH_2_PO_4_, 0.65 g/L; K_2_HPO_4_, 0.65 g/L; KCl, 0.74 g/L; NaCl, 0.58 g/L; NH_4_Cl, 0.375 g/L; MgSO_4_·7H_2_O, 0.1 g/L; CaCl_2_·2H_2_O, 0.1 g/L; 0.1 mL/L of a trace element mixture containing (per litre): iron(II) sulfate heptahydrate (FeSO_4_·7H_2_O), 1 g; zinc chloride (ZnCl_2_), 0.07 g; manganese(II) chloride tetrahydrate (MnCl_2_·4H_2_O), 0.1 g; boric acid (H_3_BO_3)_, 0.006 g; calcium chloride hexahydrate (CaCl_2_·6H_2_O), 0.13 g; copper (II) chloride dihydrate (CuCl_2_·2H_2_O), 0.002 g; nickel (II) chloride (NiCl_2_·6H_2_O), 0.024 g; sodium molybdate (Na_2_MoO_4_·2H_2_O), 0.036 g; cobalt(II) chloride (CoCl_2_·6H_2_O), 0.238 g and vitamins.

The synthetic wastewater simulated reject wastewater during conventional wastewater treatment which usually has high P concentration. After the start-up period, four operational parameters (COD concentration, anolyte and catholyte volume and cathode dissolved oxygen concentration) were monitored in order to determine their impacts on energy generation and P recovery.

### 2.3. Analytical Methods

The voltage across the external resistance was recorded every 15 min using ADC-20 data logger system (Pico Technology, Saint Neots, UK), which was connected to a computer. Based on the recorded voltage, current (I) and power (P = IV) were calculated according to Ohm’s law. The current density and power density were calculated by dividing the current and the power by the anode area (25 cm^2^) Coulombic efficiency (CE) was calculated by integrating the measured current relative to the theoretical current previously described by Equation (3) [11]. The polarization curves were determined by varying the external resistance from 10,000 to 10 Ω with an interval of 15 min to obtain a stable voltage reading:

(3) $$CE=\frac{M\int_{0}^{t}I\;dt}{Fbv_{An}\Delta COD\;}$$

where M = 32, the molecular weight of oxygen, F is Faraday’s constant, b = 4 is the number of electrons exchanged per mole of oxygen, v_An_ is the volume of liquid in the anode compartment, and ∆COD is the difference between anode COD influent and anode COD effluent over time t_b_ (cycle duration). COD removal efficiency was calculated using Equation (4):

(4) $$COD\;removal\;efficiency=\frac{anode\;COD\;influent-anode\;COD\;effluent}{anode\;COD\;influent}\;\times 100$$

The chemical oxygen demand (COD), total nitrogen (TN), ammonium (NH_4_-N), nitrate (NO_3_), nitrite (NO_2_) and total phosphorus (TP) were measured using a DR3900 spectrophotometer (HACH, Salford, UK) according to its operating procedures. Water quality analyses were conducted to assess the wastewater treatment efficiency of the MFC. pH and oxidation-reduction potential (ORP) were recorded and monitored in real time using a multi-channel parameter data recorder (EA Instruments, London, UK). Probes for measuring pH and ORP were connected to the data logger which was connected to a personal computer.

### 2.4. Phosphorus Precipitation in MFC

The theoretical P concentration in the synthetic wastewater was approximately 8 mM at pH 7. For P precipitation, ammonium chloride and magnesium chloride solutions with the theoretical concentration of 8 mM were pumped into the cathode chamber using a peristaltic pump. The ammonium and magnesium solution was pumped with a flow rate of 6 mL/day. The flowrate was chosen to achieve a 1:1:1 molar ratio of struvite precipitation NH_4_:Mg:P. Before and after each cycle, the cathode chamber was washed with deionised water 3 times, and then cleansed and dried properly to remove any attached precipitates on the chamber walls. After each precipitation cycle, the used cathode was removed for maintenance and replaced with new ones. Cathode maintenance is essential to remove P precipitates from cathode surface, as the precipitates reduce cathode performance and dissolution treatment increases cathode performance to their initial level. The used cathodes were firstly soaked in deionised water, and thereafter in 10 mM 2-(*N*-morpholino) ethanesulfonic acid (MES) buffer adjusted to pH 5.5 with NaOH, and then finally rinsed with deionised water. At the end of each cycle, the catholyte was filtered using a 0.2 um filter membrane (Fisher Scientific, Pittsburgh, UK). The precipitate was collected and analysed by X-ray diffraction (XRD). The recovered P was calculated using Equation (5):

P_in_ − P_out_ = P _filter precipitates_ + P _attached to cathode and cell_ (5)

where P_in_ = P concentration in the cathode influent, P_out_ = P concentration in the cathode effluent, and P _filter precipitates_ = the recovered P. Prior to the start of the experiment, checks were conducted to confirm that P cannot be precipitated without an MFC. A small beaker filled with the same synthetic wastewater (pH = 7 and 8 mM of P) and the same concentration of ammonium chloride and magnesium chloride was pumped into the beaker at the same rate (6 mL/day) to achieve a molar ratio of (1:1:1). No precipitates were observed anywhere in the beaker during the operation time. Thereafter, solution pH was gradually increased using 1 M NaOH. Precipitation started to occur when the pH reached 7.58.

### 2.5. Statistical Analyses

Statistical analyses were performed using Pearson coefficient analysis to determine any correlation between the different operational conditions and the monitored parameters (anode and cathode pH, anode and cathode ORP). In particular, identifying the relationship between different operational conditions and cathode pH will lead to improved understanding of maximising P recovery in MFC.

## 3. Results and Discussion

### 3.1. Phosphorus Recovery and Electricity Generation in Dual Chamber MFC

After the start-up period, stable electricity production was achieved in the system. At the beginning of each batch (batch duration = 48 h), electricity production peaked with a current density of (324 mA/m^2^) and decreased with time until the end of each batch. The maximum power density was achieved at COD = 0.7 g/L, and the system achieved a maximum power density of 198 mW/m^2^ with a current density of 0.49 mA/m^2^.

**Figure 2:**
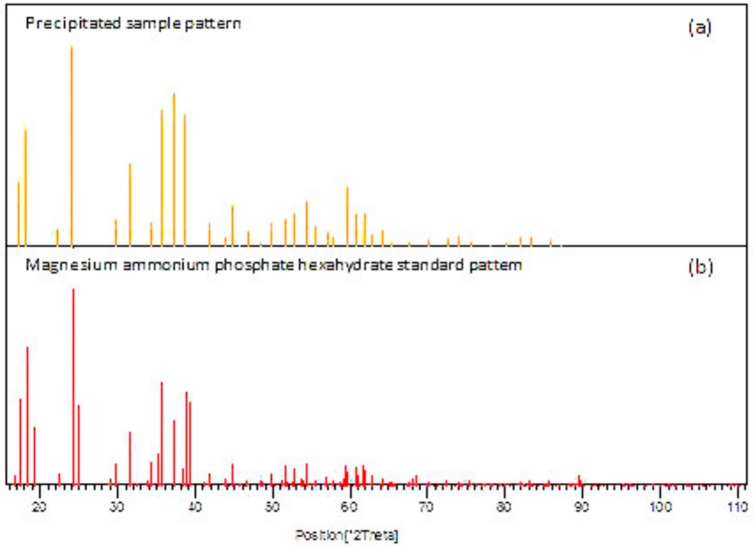
XRD patterns of precipitates in the cathode chamber, (**a**) precipitated sample; and (**b**) magnesium ammonium phosphate standard.

Due to the degradation of organic matter at the anode, electrons are released and transferred through the external resistance to the cathode. Current is generated during the transfer of electrons from anode to cathode. At the cathode, water is consumed and hydroxide is generated; this increases the pH around the cathode electrode (pH > 8). Precipitates around the cathode were observed after a short period of adding Mg and NH_4_ solution to the cathode chamber. Cathode effluent was filtered at the end of each batch and the precipitations were analysed using XRD (see Figure 2). P was recovered in the dual chamber MFC as magnesium ammonium phosphate hexahydrate (struvite) at the cathode chamber, by dosing 8 mM of NH_4_Cl and MgCl_2_ (0.76 g/L and 0.42 g/L, respectively) to achieve a molar ratio (Mg: NH_4_: P: 1:1:1).

**Figure 3:**
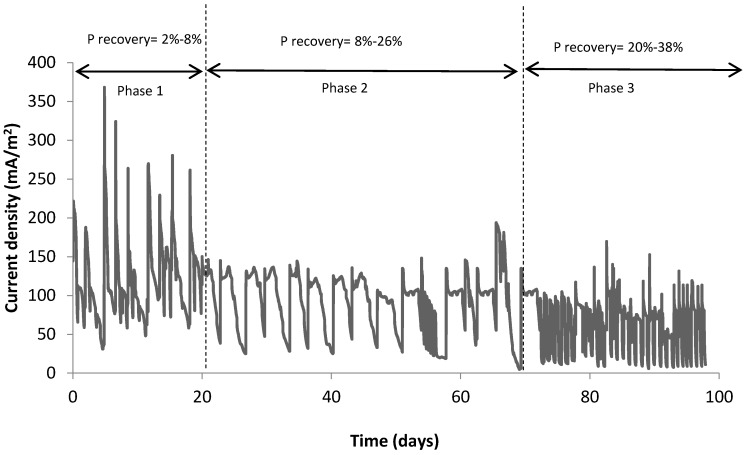
Impact of P recovery on current density.

It was observed that the greater the precipitates at the cathode, the less energy generated by the MFC. This suggests that precipitates at the cathode obstruct the mass transfer of ions and oxygen [8]. Current density was negatively affected by P precipitates at the cathode chamber, where low current density (62 mA/m^2^) was observed with high P precipitates (Figure 3). During phase 1 (COD = 0.7 g/L), the maximum current density achieved was 370 mA/m^2^ and average cathode pH was 7.4. Small amount of struvite was precipitated in the cathode chamber (2%–8% of P). In phase 2 (COD = 1 g/L), the current density decreased significantly as compared to phase 1, where average cathode pH increased to 7.8 and struvite precipitate increased from 8% to 26%.

**Figure 4:**
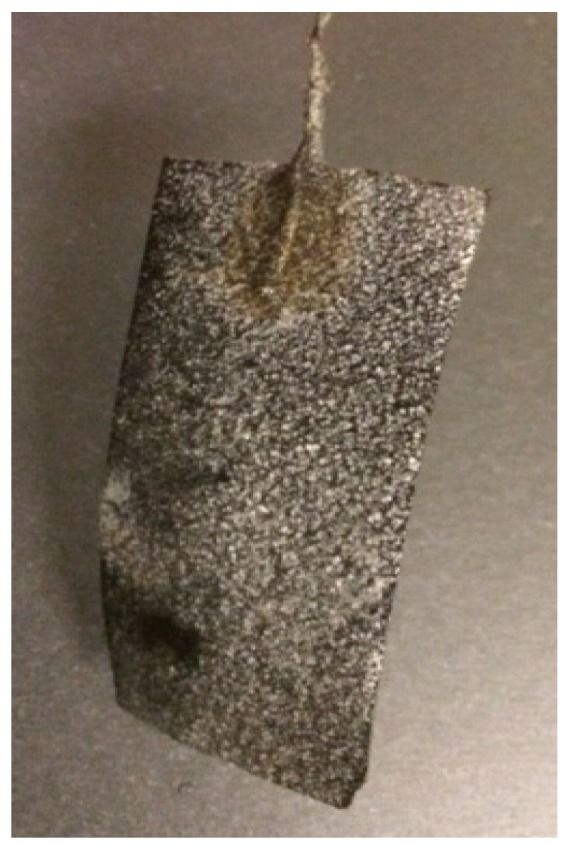
Evidence of phosphorus precipitation on the cathode.

In phase 3 (COD = 1.5 g/L), the decrease in current density was significantly more as a result of the increased amount of precipitates on the cathode electrode (Figure 4). Average cathode pH increased and peaked at 8.3. The maximum P recovery was achieved in phase 3 with a maximum recovery of 38%. These results implies that the greater the precipitates at the cathode, the less energy generated by the MFC.

### 3.2. COD Removal and Coulombic Efficiency

The system was operated at three different COD concentrations (0.7, 1.0 and 1.5 g/L) to simulate COD concentrations of reject wastewater. The anode COD removal efficiency ranged from 70% to 90%. At low COD concentration, the average removal efficiency was 90% and at high COD concentration, the average removal efficiency was 70%. Increasing influent COD concentration decreased COD removal efficiency. The average coulombic efficiency was 10% and it decreased with increasing COD concentration. Similar findings were reported by Sleutels *et al.* [12], where it was noted that increasing substrate concentration leads to decreases in the coulombic efficiency. Furthermore, increasing cathode pH due to hydroxide generation leads to a decrease in electricity generation, and this then decreases the coulombic efficiency [13]. This result demonstrates that COD concentration is an important factor for obtaining high coulombic efficiency and energy generation in MFCs. The coulombic efficiency can be improved by using a better configuration of dual chamber MFC. The type used in the current study is the H-type which is known to be a low-efficiency system due to the limited surface area for ion exchange membranes, and the long distance between electrodes [14]. In addition, the diffusion of oxygen from the cathode and from the pH and ORP probes port decrease the coulombic efficiency showing that the oxygen could work as an electron acceptor in the anode chamber.

### 3.3. Effect of Different Operational Parameters

The performance of MFCs can be influenced by different operational parameters such as: COD concentration, chamber volume and catholyte aeration flow rate. It is therefore very important to understand the impact of these parameters on MFC performance. The influence of COD concentration which acts as fuel for the bacteria, electrolyte volume and cathode air flow rate on MFCs electricity generation have been studied [13,15,16,17]. In general, the studies show that increasing COD concentration and cathode aeration leads to increased energy generation; however, the influence of these parameters on P recovery has not been investigated.

#### 3.3.1. P recovery at Different Substrate Concentrations

**Figure 5:**
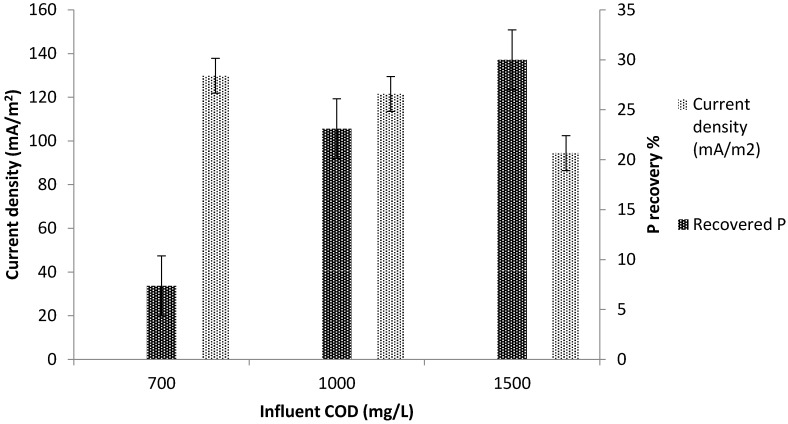
Impact of COD on current density and P recovery.

**Figure 6:**
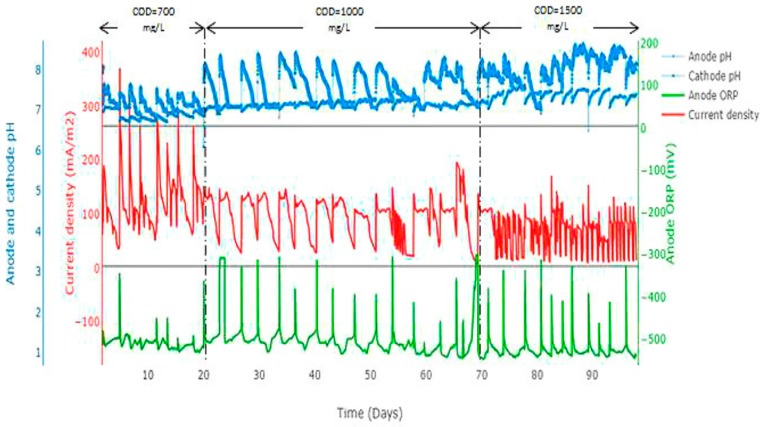
Impact of COD on current density, anode ORP, anode and cathode pH.

Different COD concentrations were used to identify the impacts of COD concentration on P recovery. The COD concentration at the anode chamber varied from 0.7 to 1.5 g/L (organic loading rate = 0.35 to 0.75 g COD/L/day). It was observed that increasing the anolyte COD leads to increase in the recovered P at the cathode (Figure 5). As the COD concentration increased from 0.7 g/L to 1.5 g/L, the recovered P increased from 7% to 38%. Similarly, as influent COD concentration increased at the anode, anode oxidation reactions increased (Figure 6). This implies that organic matter degradation increased due to substrate availability for the microorganism. As a result of the organic matter degradation, electrons are liberated and transferred from the anode to the cathode [11].

**Figure 7:**
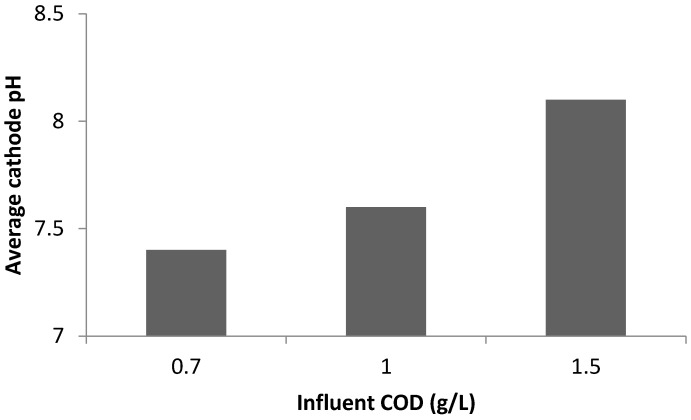
Impact of COD concentrations on cathode pH.

Increasing COD concentration leads to increase in the transfer of electrons from the anode to the cathode chamber. This implies that more electrons are available at the cathode for oxygen reduction reactions; this, in turn facilitates struvite formation. Increased oxygen reduction reactions at the cathode leads to increases in the generation of hydroxide ions; and this increased the pH to >8 at the cathode (Figure 6 and Figure 7).

It was noted that cathode pH increased as COD concentration increased (Figure 7). The average cathode pH increased from 7.4 at COD = 0.7 g/L to 8.3 at COD = 1.5 g/L. Energy generation was negatively affected by P precipitation in the cathode chamber. As COD concentration increased, more precipitates on the cathode were observed. The current density decreased with increasing COD concentration due to the amount of precipitates on the cathode surface (Figure 6).

The precipitates on the cathode covered the surface area of the cathode; and this obstructed the mass transfer of ions and oxygen. This finding is consistent with the finding of Hirooka and Ichihashi [8], which showed that the electricity generated by MFCs with precipitate was lower than that of MFCs without precipitate.

#### 3.3.2. P Recovery at Different Anode and Cathode Volumes

Electrolyte volume is another important parameter for P recovery. Three different volumes (180, 225, and 270 mL) were used to investigate the impact of anode and cathode volumes on P recovery and energy generation. The volumes were chosen based on the total volume of the chamber and the electrode surface area.

Increasing the electrolyte volume leads to increases in the P precipitated. The greater the volume of synthetic wastewater available at the anode and the cathode, the more P that can be recovered from the solution. By increasing anode chamber volume, more organic matter is offered for the organism at the anode for oxidation, and consequently; more reduction reactions occurred at the cathode. Moreover, the impact of changing chamber volume on cathode pH was not significant.

**Figure 8:**
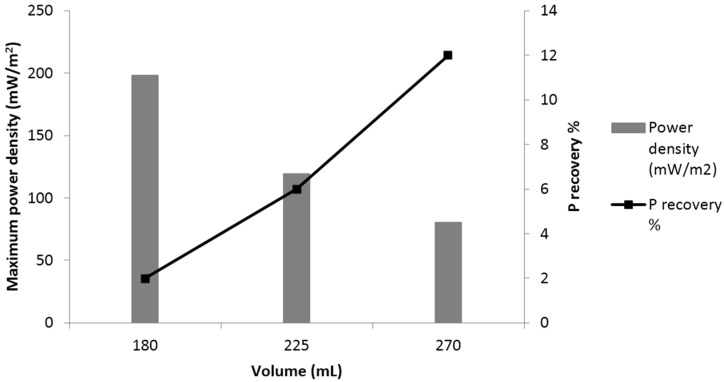
Maximum power density and P recovery % at different electrolyte volumes.

The average power density output (COD = 0.7 g/L) of the MFC under three different volumes (180, 225, 270 mL) were 25, 20 and 18 mW/m^2^, respectively. Increasing anode chamber volume from 180 mL to 270 mL leads to decreases in the average power density from 25 mW/m^2^ to 18 mW/m^2^. The decrease in power density occurred due to the amount of P precipitates in 270 mL which was greater than 180 mL. Anode and cathode volumes have an impact on energy generation, as increasing the volume leads to decreases in the power density (Figure 8).

Achieving high power density and high P recovery concurrently in the system would require further research and development as P precipitation on the cathode leads to deteriorating electricity production in the MFC; and this reduces the generated power.

#### 3.3.3. P Recovery at Different Aeration Flow Rates at the Cathode

**Figure 9:**
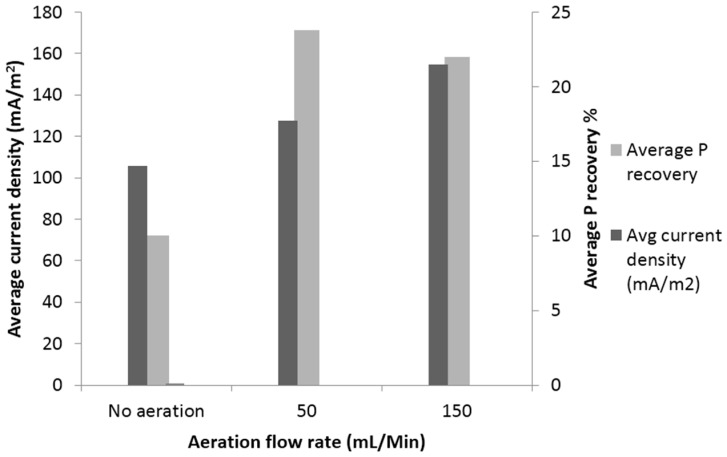
Impact of aeration flow rate on energy generation and P recovery.

Cathode aeration is an important parameter for system optimization. The microbial fuel cells were operated under controlled dissolved oxygen concentrations; different aeration flow rates were used to examine the impact of aeration on both energy generation and P recovery. Identifying the impacts of aeration flow rates on MFC performance is important for scaling up the system and assessing the operational cost. Previous studies have shown that cathode aeration has a great impact on energy generation, where the current increased as the DO concentration increased. These results show the importance of supplying the optimal amount of oxygen to obtain optimal performance of the MFC [18]. The average current density of the MFC under three different aeration flow rates (no aeration, 50 and 150 mL/min) were 92, 127 and 155 mA/m^2^, respectively. Applying aeration in the cathode chamber with a flow rate of (150 mL/min) increased the average current density by more than 40% (from 92 to 155 mA/m^2^) (Figure 9). In addition, the generated power was increased, and these were in agreement with similar findings by Mashkour and Rahimnejad [18].

**Figure 10:**
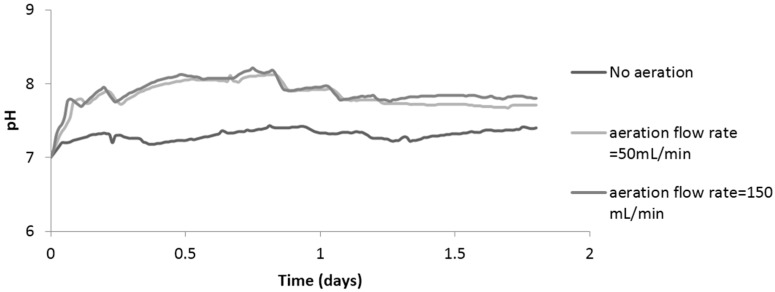
Impact of aeration flow rate on cathode pH.

With regard to P recovery, it was found that increasing aeration flow rate at the cathode leads to increase in cathode pH, where oxygen is being used as an electron acceptor and hydroxide ions are produced [10]. With (50 mL/min) flow rate, cathode pH reached 8.5, which was optimal for P recovery as struvite; whereas with no aeration, cathode pH reached 7.5 (Figure 10). The difference in P recovery between the flow rates of 50 mL/min and 150 mL/min was not significant; however, there was an increase of 25% in power density when the aeration flow rate increased from 50 to 150 mL/min. These results show that passive or low aeration cannot supply enough oxygen for the transferred protons from the anode chamber. These findings also show that cathode aeration is an influencing factor for energy generation and P recovery.

### 3.4. Statistical Analyses

The correlations between the monitored parameters and MFC performance was studied to identify the influencing factors on MFC performance for P recovery and energy generation. Table 1 shows that COD significantly affected anode pH, cathode pH and current density. The more substrate in the anode chamber, the higher the pH at the cathode. Moreover, cathode aeration has an impact on cathode pH where supplying the optimal amount of oxygen to the cathode leads to an increase in the pH. High cathode pH leads to P precipitation as struvite and deterioration in electricity generation due to the precipitates on the cathode electrode. The correlation analysis suggests that COD and cathode aeration are the key influencing factors for P recovery and energy generation. Designing the system based on these findings can help to achieve the optimal condition for energy generation and P recovery.

## 4. Conclusions

A mediator-less dual chamber microbial fuel cell was used to investigate concurrent P recovery and energy generation. Due to the high pH around the cathode, the MFC forms precipitates containing phosphorus in the catholyte and on the cathode surface. The main component of the precipitate was determined by X-ray diffraction analysis to be magnesium ammonium phosphate hexahydrate. P recovery at the cathode ranged from 1% to 38%; and correlation analysis indicated that COD and aeration flow rate are the key factors influencing P recovery and energy generation. Further studies are recommended to minimize the energy loss due to the precipitates on the cathode surface. This could be focused on a movable cathode that can be replaced once the voltage starts to decrease, or finding a specific material that prevents any particle attachment on the cathode surface.

## Figures and Tables

**Table 1:** Correlation analysis for the MFC performance.

Variable	Aeration Flow Rate	Anode pH	Cathode pH	Anode ORP	Cathode ORP	I Density
Aeration flow rate	-	-	-	-	-	-
Anode pH	0.457 *	-	-	-	-	-
Cathode pH	0.619 **	0.783 **	-	-	-	-
Anode ORP	0.111	−0.362 *	−0.330	-	-	-
Cathode ORP	0.070	0.101	0.015	0.094	-	-
I density	0.317	−0.533 **	−0.236	−0.095	−0.099	-
COD	-	0.929 **	0.803 **	−0.275	-	−0.468 **

** Correlation is significant at the 0.01 level (2-tailed); * Correlation is significant at the 0.05 level (2-tailed).

## References

[1] Cooper J., Lombardi R., Boardman D., Carliell-Marquet C. (2011). The future distribution and production of global phosphate rock reserves. Resour. Conserv. Recycl..

[2] Güney K., Weidelener A., Krampe J. (2008). Phosphorus recovery from digested sewage sludge as map by the help of metal ion separation. Water Res..

[3] Doyle J.D., Parsons S.A. (2002). Struvite formation, control and recovery. Water Res..

[4] You J., Greenman J., Melhuish C., Ieropoulos I. (2015). Electricity generation and struvite recovery from human urine using microbial fuel cells. J. Chem. Technol. Biotechnol..

[5] Cusick R.D., Ullery M.L., Dempsey B.A., Logan B.E. (2014). Electrochemical struvite precipitation from digestate with a fluidized bed cathode microbial electrolysis cell. Water Res..

[6] Jaffer Y., Clark T., Pearce P., Parsons S. (2002). Potential phosphorus recovery by struvite formation. Water Res..

[7] Cusick R.D., Logan B.E. (2012). Phosphate recovery as struvite within a single chamber microbial electrolysis cell. Bioresour. Technol..

[8] Hirooka K., Ichihashi O. (2013). Phosphorus recovery from artificial wastewater by microbial fuel cell and its effect on power generation. Bioresour. Technol..

[9] Ichihashi O., Hirooka K. (2012). Removal and recovery of phosphorus as struvite from swine wastewater using microbial fuel cell. Bioresour. Technol..

[10] Kelly P.T., He Z. (2014). Nutrients removal and recovery in bioelectrochemical systems: A review. Bioresour. Technol..

[11] Logan B.E., Hamelers B., Rozendal R., Schröder U., Keller J., Freguia S., Aelterman P., Verstraete W., Rabaey K. (2006). Microbial fuel cells: Methodology and technology. Environ. Sci. Technol..

[12] Sleutels T.H., Darus L., Hamelers H.V., Buisman C.J. (2011). Effect of operational parameters on coulombic efficiency in bioelectrochemical systems. Bioresour. Technol..

[13] Cheng S., Logan B.E. (2011). Increasing power generation for scaling up single-chamber air cathode microbial fuel cells. Bioresour. Technol..

[14] Zhang F., He Z. (2012). Simultaneous nitrification and denitrification with electricity generation in dual-cathode microbial fuel cells. J. Chem. Technol. Biotechnol..

[15] Aelterman P., Versichele M., Marzorati M., Boon N., Verstraete W. (2008). Loading rate and external resistance control the electricity generation of microbial fuel cells with different three-dimensional anodes. Bioresour. Technol..

[16] Jadhav G., Ghangrekar M. (2009). Performance of microbial fuel cell subjected to variation in pH, temperature, external load and substrate concentration. Bioresour. Technol..

[17] Nam J.Y., Kim H.W., Lim K.H., Shin H.S. (2010). Effects of organic loading rates on the continuous electricity generation from fermented wastewater using a single-chamber microbial fuel cell. Bioresour. Technol..

[18] Mashkour M., Rahimnejad M. (2015). Effect of various carbon-based cathode electrodes on the performance of microbial fuel cell. Biofuel Res. J..

